# Halocins and C_50_ Carotenoids from Haloarchaea: Potential Natural Tools against Cancer

**DOI:** 10.3390/md22100448

**Published:** 2024-09-29

**Authors:** Rosa María Martínez-Espinosa

**Affiliations:** 1 Biochemistry and Molecular Biology and Edaphology and Agricultural Chemistry Department, Faculty of Sciences, University of Alicante, Ap. 99, E-03080 Alicante, Spain; rosa.martinez@ua.es; Tel.: +34-965-903-400 (ext. 8841); 2 Multidisciplinary Institute for Environmental Studies “Ramón Margalef”, University of Alicante, Ap. 99, E-03080 Alicante, Spain

**Keywords:** haloarchaea, halocins, microhalocins, C_50_ carotenoids, bacterioruberin (BR), bisanhydrobacterioruberin (BABR), monoanhydrobacterioruberin (MABR), immunomodulation, antitumoral activity

## Abstract

Haloarchaea are a group of moderate and extreme halophilic microorganisms, belonging to the Archaea domain, that constitute relevant microbial communities in salty environments like coastal and inland salted ponds, marshes, salty lagoons, etc. They can survive in stress conditions such as high salinity and, therefore, high ionic strength, high doses of ultraviolet radiation (UV), high temperature, and extreme pH values. Consequently, most of the species can be considered polyextremophiles owing to their ability to respond to the multiple extreme conditions characterizing their natural habitats. They cope with those stresses thanks to several molecular and metabolic adaptations. Thus, some of the molecules produced by haloarchaea show significantly different biological activities and physicochemical properties compared to their bacterial counterparts. Recent studies have revealed promising applications in biotechnology and medicine for these biomolecules. Among haloarchaeal biomolecules, rare natural pigments (C_50_ carotenoids) and small peptides called halocins and microhalocins have attracted attention worldwide due to their effects on animal and human commercial tumoral cells, apart from the role as antibiotics described for halocins or the immunomodulatory activity reported from C_50_ carotenoids like bacterioruberin. This review summarizes recent knowledge on these two types of biomolecules in connection with cancer to shed new light on the design of drugs and new therapies based on natural compounds.

## 1. Introduction

Halophilic microorganisms can be found in all three domains of life: Archaea, Bacteria, and Eukarya. They inhabit saline environments, mainly those showing total salt concentrations above 1 M. Based on the optimum salinity requirements, halophiles are usually classified into three major groups: slight halophiles (0.34 to 0.85 M), moderate halophiles (0.85 to 3.4 M), and extreme halophiles (3.4 M to saturation point) [1]. Microorganisms showing moderate or extreme halophilic profiles are mainly bacterial and archaeal members. Specifically, in very saline ecosystems (2–4 M NaCl), halophilic archaea (haloarchaea) of the families *Halobacteriaceae* and *Haloferacaceae* usually constitute the most abundant microbial populations apart from β-carotene-rich species of the unicellular green algal genus *Dunaliella* and bacteria of the genus *Salinibacter* [2,3].

Haloarchaea are more widely spread than initially thought and have evolved to reach several molecular and metabolic adaptations to survive different stresses, among which high UV radiation and salt stress are the most significant [4]. Thus, some haloarchaea are considered polyextremophiles owing to their ability to respond to multiple extreme conditions: (i) they can adjust to osmotic stress and survive with low water activity and desiccation thanks to a salt-in strategy (KCl is accumulated inside cells with the help of protein transport and ion pumps) [4] and low-salt-in strategy (compatible and low-molecular-weight solutes are synthesized by the cells to be more adapted to osmotic stress) [5]; (ii) haloarchaeal cells tolerate different temperature variations thanks to heat shock proteins (e.g., chaperones and chaperonins) that contribute to the folding or unfolding of proteins at extreme temperatures [6]; (iii) the formation of photoproducts and pyrimidine dimers in DNA due to high UV doses is compensated by a photoreactivation process that can remove these lesions. In these processes, biomolecules like enzymes (i.e., photolyases) and carotenoids are essential [4]. (iv) Molecular adaptations affecting protein composition and structure include a high content of acidic amino acids on the surface and less hydrophobic interactions owing to the limited content of hydrophilic amino acids such as lysine to ensure the presence of active proteins and enzymes in the cytoplasm and extracellular media characterized by high ionic strength due to high KCl or NaCl concentrations [7].

The consequence of all these molecular adaptations is that several molecules are unique from a biochemical point of view compared to their bacterial counterparts. Many studies reported during the last two decades using haloarchaeal species as model microorganisms have revealed that these microorganisms are excellent natural sources of high-value biomolecules like enzymes, antimicrobials, carotenoids (pigments), compatible solutes, lipids, bioplastics, antiadhesives, and biofuels [8,9]. Because of this, haloarchaea have caught the attention of many industries related to medicine, nutraceuticals, pharmaceuticals, laundry, food, coloring, chemical formulations, and many others yet to be explored [8,9,10,11].

By the end of the last century, it was considered that studies of archaea were of great difficulty compared to those of bacterial species due to technical limitations regarding the production of mutants or the upscaling of biotechnologically based processes. Fortunately, advances in basic and applied research on the biochemistry, physiology, and molecular biology of haloarchaea, as well as on omics-based strategies, make currently possible the use of haloarchaea as cellular factories to overproduce high-market-value biomolecules like enzymes, carotenoids, or bioplastics [8,12,13].

Related to potential applications in medicine of biomolecules synthesized by haloarchaea, rare carotenoids and halocins/microhalocins stand out for their high antioxidant capacity or use as antibiotics, respectively, and in both cases, for their antitumor activity. This review summarizes current knowledge about two types of biomolecules almost exclusively produced by haloarchaea for which antitumoral activity has been described: C_50_ carotenoids (also termed rare carotenoids: bacterioruberin (BR), bisanhydrobacterioruberin (BABR), monoanhydrobacterioruberin (MABR)) as well as microhalocins and halocins.

## 2. Halocins: Description and Potential Uses as Antibiotics and Antitumoral Molecules

Archaeocins are antibiotic small peptides sourced from archaea, being found widely amongst haloarchaea (termed halocins) and more recently from the *Sulfolobus* genus (sulfolobicins) [14]. These peptides are secreted into the environment to kill or inhibit the metabolism or even the growth of other microorganisms that occupy the same niche, thus competing for nutrients, oxygen, etc. [15,16]. The production of halocins seems to be a universal trait among haloarchaea, and they would act similarly to bacteriocins from bacteria [17,18]. Despite the relevant role of halocins in halophilic ecosystems (i.e., modulating microbial interactions), only a few halocin-producing species have been studied in detail. For example, the synthesis of halocin C8 (probably the best-characterized halocin) in the genera *Natrinema*, *Haloterrigena*, *Haloferax*, and *Halobacterium* has been confirmed. Additionally, putative gene sequences coding for halocin C8 have also been reported in *Halopiger*, *Halostella*, *Halorussus*, and unclassified Archaea [19]. The size of the currently described micro/halocins (Table 1) ranges from 3.6 kDa to 35 kDa. Based on de molecular mass, halocins have been traditionally classified into two main groups: (i) halocins showing a molecular mass > 10 kDa (examples, halocin H1 (HalH1) and H4 (HalH4), which range from 30 to 35 kDa) and (ii) microhalocins, which are peptides showing a molecular mass < 10 kDa (examples, halocin S8 (HalS8), R1 (HalR1), C8 (HalC8), U1 (HalU1), H6 (HalH6), Sech7a and Sech10). All halocins described up to now are hydrophobic, retaining activity without salt. Moreover, most halocins can be stored at 4 °C and are tolerant of heat and organic solvents. A summary of the halocins described to date and their properties can be found in Table 1.

Several studies confirmed that haloarchaea produce halocins during the transition from an exponential to a stationary growth phase, showing no decline in halocin production levels during the latter [32,36]. Most of the halocins described show a broad spectrum of action as antimicrobial molecules, meaning that the target can be haloarchaea but also Gram-positive and Gram-negative bacteria and even eukaryotic cells [18,37]. For instance, halocins produced by *Natrinema* spp. impair the growth of a diversity of haloarchaea, including those from the genera *Halorubrum*, *Halobacterium, Haloarcula*, *Haloferax*, *Natronobacterium*, and *Natronomonas* [21,35,36]. Microhalocins like S8a, GN101, and TuA4 (from Euryarchaeota halophilic strains) affect a variety of haloarchaeal genera (kingdom Euryarchaeota), and exhibit cross-kingdom toxicity, inhibiting or killing members of the hyperthermophilic crenarchaeal genus *Sulfolobus*. HalR1 produced by *Halobacterium* sp. can inhibit *Methanosarcina thermophiles* [38]. Other studies have proven the antimicrobial effect of halocins against Gram-positive and Gram-negative human pathogens [39]. Additionally, research on halocin H6 confirmed inhibitory activity in the Na+/H+ exchange (NHE) in eukaryotic cells [40].

The mechanism of action of most halocins remains undiscovered; unlike the antibiotics produced by bacteria and eukaryotes, microhalocins are not cationic and possess many neutral residues. Notwithstanding, it has been suggested that some of them (H4, HA1, HA3, Sech7a, H6, C8, and SH10) produce an osmotic imbalance that results in swelling, which eventually causes cell lysis of sensitive microorganisms or even in commercial tumoral cells [32]. Moreover, recent studies have attributed them to other physiological functions like DNA uptake [14].

Although a significant number and variety of halocins have been isolated and described, most of them have not been fully sequenced, and therefore, it is hard to detect their presence in other taxa different from those from which halocins have been initially described. Nowadays, the best-described halocin at the molecular level is C8. The halocin C8 gene (halC8) encodes the 283-amino-acids-long C8 precursor protein “ProC8”. This protein is exported outside the cell via the Tat pathway where it matures, resulting in the immune peptide HalI (243 amino acids) and the mature halocin C8 (76 aa) [21,22]. Table 2 displays information on genes coding for halocin C8 in haloarchaea.

A few studies have demonstrated that among the important uses of halocins, the following can be highlighted apart from antimicrobial activity: preservation of salted food products and brine-cured hides in leather industries, protection of tanned skin, and protection of the myocardium from ischemia and reperfusion injury, as well as from life-threatening diseases such as cardiac arrest and cancers [20]. Regarding cancer, the most likely hypothesis suggests that the osmotic imbalance produced by halocins could be the reason behind the potential antitumoral activity of halocins. Nevertheless, more accurate studies are required to confirm this antitumoral activity using tumoral cells exposed to halocins. Consequently, there is a great potential for applications in the biomedical industry, including cancer treatments and the use of halocins as tools promoting DNA uptake as part of molecular therapies [14].

## 3. C_50_ Carotenoids: Description and Potential Uses against Cancer

Carotenoids are widespread natural pigments well characterized in plants, algae, fungi, and bacteria. However, the knowledge of haloarchaeal carotenoids is poor compared to natural pigments from other living beings. The first reported studies on these compounds date back to the 1970s, using *Halobacterium cutirubrum* as a model microorganism. The studies mainly conducted during the last two decades have confirmed that the major carotenoid produced by the haloarchaeal cells is the rare C_50_ called bacterioruberin (BR), followed by monoanhydrobacterioruberin (MABR), at the expense of lycopene and bisanhydrobacterioruberin (BABR), both MABR and BABR being precursors of BR [41,42]. Other carotenoids have been identified in haloarchaeal carotenoid extracts (including β-carotene, lycopene, and some xanthophylls); however, it is currently assumed that the most abundant in all haloarchaeal carotenoid extracts characterized so far is BR, a natural pigment responsible for biological activities of high interest and potential applications in biotechnology and biomedicine [43]. BR consists of a primary conjugated isoprenoid chain that contains 13 conjugated double bonds and four hydroxyl groups arising from the terminal ends. Table 3 displays the chemical structure, chemical formula, and complete name of BR and its precursors.

Some studies confirmed that a few extremophilic bacterial species like the Antarctic psychrotrophic bacterium *Micrococcus roseus* and *Arthrobacter* species are also BR producers [44,45]; however*,* it is well accepted that BR is produced almost exclusively by haloarchaea. The fact that BR-producing bacteria are extremophilic, together with the polyextremophilic character of BR-producing haloarchaea, reflects the important role that BR plays in adaptation to life in extreme conditions (especially high ionic strength, high oxidative stress, and low water availability), as has already been widely described in the literature [12,43,46].

A few studies have explored the synthesis of carotenoids (carotenogenesis) in haloarchaea from bioinformatics to biochemistry and molecular biology points of view. The common feature found in these studies reveals that haloarchaeal carotenogenesis depends on the mevalonate pathway to produce the carotenoid precursor isopentenyl pyrophosphate. Then, it is converted into trans-phytoene, which leads to ζ-carotene further converted to neurosporene. Neurosporene is transformed into lycopene, from which most carotenoids, including BR, derive [47]. BR fits in between the glycerolipids, forming the bilayer of the membrane cells, with the hydroxyl group facing outwards and inwards. Due to the location of BR and its derivatives, these natural pigments play a pivotal role in membrane stability, acting as a protection mechanism against the harsh conditions usually present in the natural environment of these extremophilic microorganisms, such as high oxidative and osmotic stress and elevated radiation [44,46,47,48,49]. BR protects cells from oxidative damage by acting as an antioxidant thanks to the electron transport between the pairs of conjugated double bonds. Since bacterioruberin presents a longer hydrocarbon chain and a higher number of conjugated double bonds than other carotenoids, such as β-carotene (C_40_ carotenoid, nine conjugated double bonds), it has an extraordinary scavenging activity, which is essentially the biological activity that makes BR of interest in several industrial and biomedical sectors due to its high antioxidant activity [12,43,46,49,50,51]. Based on the chemical composition and structure of BR, it was initially assumed that this natural carotenoid has strong antioxidant properties, as has been later quantified when compared to one of the most marketed carotenoids, β-carotene [51,52].

In connection with the antioxidant activity of BR, recent studies have described the anti-inflammatory, antitumoral, and immunomodulatory benefits of BR in human commercial cell lines representative of different pathologies. For instance, a study carried out with BR isolated from the haloarchaeon *Halorubrum tebenquichense* suggested that the carotenoid in combination with dexamethasone (Dex) in ultra-small macrophage-targeted nanoparticles could act as a potential intestinal repairing agent [53]. In another study, a carotenoid extract rich in BR and C_18_ fatty acids from *Haloarcula* sp. was used as the source of carotenoid extract to monitor its effect on lipopolysaccharide (LPS)-stimulated macrophages, which resulted in a reduction in ROS production, a decrease in the pro-inflammatory cytokines TNF-α and IL-6 levels, and an upregulation of the factor Nrf2 and its target gene heme oxygenase-1 (HO-1). The main conclusion was that this extract could act as a therapeutic agent in the treatment of oxidative stress-related inflammatory diseases [54].

Considering these results, more recently, some research groups worldwide have analyzed the effect of BR on tumoral cells (mainly cell lines). Thus, it has been demonstrated that carotenoid-rich extracts from a haloarchaeal strain (M8) (with a total concentration of carotenoids ranging between 0.2 and 1.5 μM) reduced hepatoma cell line (HepG2) viability up to 50% in a concentration-dependent manner. In addition, hepatoma cells treated with haloarchaeal carotenoids were less sensitive to oxidative stress generated by H₂O₂, thus exerting a protective effect [55]. The antiproliferative effect on hepatoma cells was also reported for extracts isolated from *Halogeometricum limi* and *Haloplanus vescus* [51]. The anticancer effect of *Natrialba* sp. M6 carotenoid extract was reported again for hepatoma cells (HepG2) as well as for other types of cancer cell lines, including Caco-2 (colon cancer), MCF-7 (breast cancer), and HeLa (cervical cancer) [56]. In the case of MCF-7 commercial cell lines, a real-time PCR technique was used to monitor the expression of genes specific for apoptosis, in the presence or absence of BR-rich carotenoid extract. Both early and late apoptosis were increased significantly by about 10% and 39%, respectively, due to the upregulation of the expression of some genes (mainly, CASP3, CASP8, BAX) in the MCF-7 cell line. In contrast, the expression of genes like MKI67 and SOX2 were significantly downregulated in the treated MCF-7 cell line. The antiproliferative effect on breast cancer cell lines has been explored in other studies using BR extracts isolated from species belonging to *Haloarcula* and *Haloferax* genera [43,57,58]. In particular, *Haloferax mediterranei* carotenoid extracts reduced cell adhesion, viability, diameter, and concentrations in cell lines representative of the four well-defined subtypes of breast cancer (Luminal A, Luminal B, HER2-enriched and triple-negative), all of them exposed to different concentrations of the BR-rich extract [57]. In the case of *Haloarcula*, carotenoid extracted from strain A15 had the most potent cytotoxic effect on the breast cancer MCF-7 cell line (IC_50_ = 0.0645 mg/mL) [58]. One of the main discussions that are currently the subject of analysis is whether, in these extracts rich in BR, the mentioned biological activities are due to BR, its precursors (which in minimal concentrations are also present), or the presence of all of them (bacterioruberin, monoanhydrobacterioruberin, and bisanhydrobacterioruberin).

In conclusion, the observed antiproliferative effects of BR-rich extracts from various haloarchaeal strains, notably on hepatoma and breast cancer cell lines, suggest its potential as a valuable candidate for novel anticancer therapies. However, to translate these findings into clinically relevant interventions, further investigations are necessary to elucidate the underlying molecular mechanisms driving the anticancer properties of BR and its precursors. Additionally, comprehensive studies are needed to assess potential interactions between BR and current anticancer drugs, ensuring their compatibility and optimizing the therapeutic outcomes. It is important to acknowledge the limitations associated with the use of carotenoids, including challenges in identifying optimal doses and potential variations in bioavailability. Moreover, while current preliminary studies may focus on treatment perspectives, clinical investigations with other carotenoids often adopt a preventive approach, which limits the accuracy of direct comparisons. Addressing these complexities will be essential for advancing our understanding of BR’s therapeutic potential and developing effective strategies for cancer management.

## 4. Future Perspectives

Considering the applications that halocins and BR could have in the medical and pharmaceutical field, successful processes aiming at the purification and characterization of these molecules have been developed during the last two decades. Once the molecules were characterized from a biochemical point of view, those studies have been complemented with bioinformatic-based analysis to identify the relevant genes and pathways involved in synthesizing both types of molecules. In this context, carotenogenesis in haloarchaea was fully elucidated, and a few groups all over the world have obtained transformants and mutants to optimize the production of bacterioruberin. Unfortunately, the bioinformatic and genomic work regarding halocins is scarce compared to haloarchaeal carotenoids, thus becoming one of the main challenges to be overseen shortly. To contribute to the advancement of basic and applied knowledge about these molecules, the main milestones achieved and the challenges to be faced concerning each of these two types of molecules are highlighted below.

Main milestones: carotenogenesis in haloarchaea has been deeply described using bioinformatics and genomics, and mutants have been obtained to overproduce BR at the laboratory scale [47,50]. Some recent studies have demonstrated that the scalability of haloarchaea cultivation to produce BR is feasible [12]. As an example, circular economy-based production has been described using waste materials from several industries as raw materials for haloarchaeal growth and BR production [12,48,49]. Consequently, the large-scale production of BR will be possible shortly (currently there is at least one company already commercializing BR in Europe: HALOTEK Applied Biotechnologies). Green chemistry-based processes for the extraction of BR have also been optimized (for example, using Eutectic Solvents), thus addressing the environmental and economic concerns of developing haloarchaeal biomolecules for pharmaceutical use [59]. Further, the biological activities of BR as an antioxidant, antitumoral, immunomodulatory, antilipidemic, and antiglycemic have been extensively investigated [43,46,50,51,52,53,56,57,60].

Challenges: so far, the overproduction of halocins by haloarchaeal mutant strains (or even using heterologous overexpression approaches) has been poorly explored, and the mechanisms of action of halocins remain unknown. On the other hand, it is necessary to develop more research with halocins to accurately describe the real spectrum of the biological activities of these molecules that could have an impact on medicine. Consequently, genomic and more biochemical work on halocins should be a trending topic for research during the next few years. Regarding BR, the molecular mechanisms of action are also poorly described, apart from the fact that apoptosis of tumoral cells is observed when tumoral cell lines are exposed to BR. The potential for halocins and BR to reduce side effects or improve the efficacy of conventional treatments of cancer and immune system-related diseases must be a focus of global attention shortly as a key area of exploration. Finally, the up-scaled production of halocins is far from being achieved unless efficient strains producing these molecules arise shortly.

## 5. Conclusions

Several haloarchaeal species and some of their molecules could provide innovation and benefits in a wide range of applications in biomedicine, medicine, and pharmaceuticals (apart from food processing and textiles). In the case of halocins and BR, recent studies have demonstrated their powerful activities as antimicrobial or antitumoral/immunomodulatory/anti-inflammatory molecules, respectively. These findings offer new approaches and strategies to define new drug formulations or drug immobilization as part of the treatments of pathologies related to the immune system, microbial infections, and cancer, among others. By promoting research on haloarchaeal biomolecules, it is possible to uncover novel applications for these promising C_50_ carotenoids. Furthermore, the cultivation of haloarchaea and green-based processes to isolate the molecules make haloarchaea attractive subjects for research and the development of sustainable processes aiming at the production of natural pigments with a wider spectrum of applications, all following circular economy-based processes.

## Figures and Tables

**Table 1:** Halocins and microhalocins isolated from haloarchaea. ND: non-determined.

Type	Species/Strains	Characterization Parameters: Molecular Weight, Thermostability, Salt Dependence	References
A4	Strain TuA4	7.4 kDa, >100 °C, ND	[20]
C8	*Natrinema* sp. strain AS7092 (formerly *Halobacterium* sp. AS7092)	6.3 kDa, >100 °C, ND	[16,21,22]
	*Natrinema* sp. *RNS21*	7.4 kDa, ND, ND	[23]
G1	*Halobacterium* strain GRB	ND, ND, ND,	[20]
H1	*Haloferax mediterranei* M2a (Xai3)	31 kDa, <50 °C, yes	[24,25]
H2	Haloarchaeon Gla2.2	ND, ND, ND	[20]
H3	Haloarchaeon Gaa12	ND, ND, ND	[20]
H4	*Haloferax mediterranei* R4	34.9 kDa, <60 °C, partially	[26,27]
H5	Haloarchaeon Ma2.20	ND, ND, ND	[20]
H6/H7	*Haloferax gibbonsii Ma2.39*	32 kDa, <90 °C, no	[28]
HA1	*Haloferax larsenii* KPS1	∼14 kDa, <121 °C, ND pH 4.0–12.0	[29]
HA3	*Haloferax larsenii* NCIM5678	13 kDa, ND, ND	[30]
HA4	*Haloferax larsenii* (HA4)	~14 kDa, <100 °C, ND pH 2.0–10.0,	[31]
R1	*Halobacterium* strain GN101	3.8 kDa, <93 °C, no	[32]
S8	Strain S8a	3.6 kDa, >100 °C, no	[33]
Sech7a	*Haloferax mediterranei* Sech7a	10.7 kDa, <80 °C, yes	[34]
SH10	*Natrinema* sp. BTSH10	20 kDa, <50 °C, ND	[35]

**Table 2:** Genes coding for halocin C8 identified in haloarchaeal genomes. The search was conducted through UniProt (www.uniprot.org, accessed on 15 July 2024) (90% similarity with HalC8 (P83716)). The number of amino acids for each halocin is also indicated.

Entry	Gene Name	Organism	Nº Amino Acids
A0A1W6ALE4	*halC8*	*Haloterrigena thermotolerans*	283
L9ZDM4	*C485_14015*	*Natrinema altunense* JCM 12890	248
A0A1W6ALE2	*halC8*	*Natrinema versiforme*	217
A0A0K0KG39	*proC8*	*Natrinema* sp. SSI3	283
A0A0K0KFP1	*proC8*	*Natrinema* sp. SI14	283
A0A1W6ALD3	*halC8*	*Haloterrigena turkmenica*	283
A0A1W6ALD1	*halC8*	*Natrinema ejinorense*	283
A0A1W6ALB1	*halC8*	*Haloterrigena jeotgali*	283
A0A1W6ALC8	*halC8*	*Natrinema altunense*	283
A0A0K0KFY8	*proC8*	*Natrinema* sp. SI4	283
A0A0K0KGM5	*proC8*	*Natrinema* sp. SWI6	283
A0A0K0KFP9	*proC8*	*Natrinema* sp. SWI15	283
A0A1W6ALC4	*halC8*	*Natrinema salaciae*	283

**Table 3:** Structures and common and scientific names of bacterioruberin (BR) and its precursors, adapted from [43].

Common Name and Chemical Formula	Chemical Structure (Stereoisomers)
Bacterioruberin C_50_H_76_O_4_	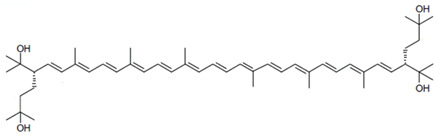 (2*S*,2′*S*)-2,2′-bis(3-hydroxy-3-methylbutyl)-3,4,3′,4′-tetradehydro-1,2,1′,2′-tetrahydro-γ,γ-carotene-1,1′-diol
Monoanhydrobacterioruberin C_50_H_74_O_3_	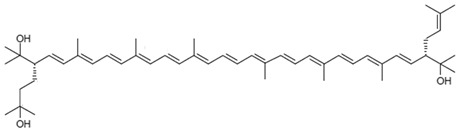 (3*S*,4*E*,6*E*,8*E*,10*E*,12*E*,14*E*,16*E*,18*E*,20*E*,22*E*,24*E*,26*E*,28*E*,30*S*)-30-(2-hydroxypropan-2-yl)-2,6,10,14,19,23,27,33-octamethyl-3-(3-methylbut-2-en-1-yl)tetratriaconta-4,6,8,10,12,14,16,18,20,22,24,26,28-tridecaene-2,33-diol
Bisanhydrobacterioruberin C_50_H_72_O_2_	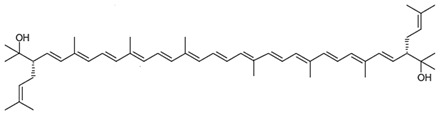 (3*S*,4*E*,6*E*,8*E*,10*E*,12*E*,14*E*,16*E*,18*E*,20*E*,22*E*,24*E*,26*E*,28*E*,30*S*)-2,6,10,14,19,23,27,31-octamethyl-3,30-bis(3-methylbut-2-en-1-yl)dotriaconta-4,6,8,10,12,14,16,18,20,22,24,26,28-tridecaene-2,31-diol
